# Risk factors and baseline cognition. Preliminary results from the LatAm‐FINGERS cohort

**DOI:** 10.1002/alz.091921

**Published:** 2025-01-09

**Authors:** Nicolas Corvalan, Greta Keller, Ezequiel Ignacio Surace, Ismael Luis Calandri, Sonia Maria Dozzi Brucki, Rosa Maria Salinas‐Contreras, Claudia Kimie Suemoto, Lina M Velilla, Monica Sanches Yassuda, Ricardo Allegri, Paulo Caramelli, Francisco Lopera, Ricardo Nitrini, Ana Luisa Sosa, Gustavo Sevlever, Lucia Crivelli, LatAm‐FINGERS Consortium

**Affiliations:** ^1^ Fleni, CABA, Buenos Aires Argentina; ^2^ Fleni, Buenos Aires, CABA Argentina; ^3^ Fleni, Buenos Aires Argentina; ^4^ Fleni, Buenos Aires, Buenos Aires Argentina; ^5^ University of São Paulo School of Medicine, São Paulo, São Paulo Brazil; ^6^ Dementias Laboratory, National Institute of Neurology and Neurosurgery, Mexico City, DF Mexico; ^7^ University of São Paulo Medical School, São Paulo, São Paulo Brazil; ^8^ Neurosciences Group of Antioquia, University of Antioquia, Medellín Colombia; ^9^ University of São Paulo, SAO PAULO, SAO PAULO Brazil; ^10^ Faculty of Medicine ‐ Universidade Federal de Minas Gerais, Belo Horizonte Brazil; ^11^ Grupo de Neurociencias de Antioquia, Universidad de Antioquia, Medellin Colombia; ^12^ Department of Cognitive Neurology, Montañeses, Buenos Aires Argentina

## Abstract

**Background:**

LatAm‐FINGERS ‐ the first non‐pharmacological multicenter randomized clinical trial in Latin America ‐ is a valuable opportunity to study lifestyle in a heterogeneous and multiethnic population exposed to a large number of cardiovascular risk factors. Our aims are to study the risk distribution in the LatAm‐FINGERS cohort and to explore the relationship between LIfestyle for BRAin Health (LIBRA) and cognition.

**Method:**

We calculated the risk of dementia using the LIBRA score in the entire cohort (n = 1200). LIBRA consists of a weighted sum score (higher scores indicating greater dementia risk) of 12 modifiable risk and protective factors for cognitive decline. Cognition was assessed using the Latin America Neuropsychological Test Battery (LatAm‐NTB). We constructed mixed‐effects linear regression models to examine the relationship between the LIBRA and cognition as a fixed effect, with country as a random effect.

**Result:**

The LIBRA ranged from ‐4.5 to 10, with a mean of 2.39 (±2.75). Men had significantly higher LIBRA scores than women (w = 125887, p = .003). Those with more education (>9 years) scored significantly lower in LIBRA compared to those with less education (w = 57458, p < .001), possibly due to the established association between education and health outcomes. No differences were found in LIBRA between individuals with and without APOE4 (w = 17475, p = 0.57). A significant relationship was observed between LIBRA and global cognition measured by LatAm‐NTB (β = ‐0.03, std. error = 0.004, t value = ‐9.28, p < .001). There was a relationship between LIBRA and memory (β = ‐0.05, std. error = 0.007, t value = ‐7.06, p < .001), executive functions (β = ‐0.02, std. error = 0.004, t value = ‐5.49, p < .001), and processing speed composites (β = ‐0.01, std. error = 0.005, t value = ‐3.30, p < .001).

**Conclusion:**

Our results shed light on sex differences, revealing that men tend to exhibit higher LIBRA. The negative relationship between higher levels of education and LIBRA underscores the protective role of education on cognition. This highlights the significance of LIBRA as a useful risk composite for elucidating cognitive outcomes within the LatAm‐FINGERS cohort, characterized by its ethnic and cultural diversity.

